# *ARID1A, ARID1B*, and *ARID2* Mutations Serve as Potential Biomarkers for Immune Checkpoint Blockade in Patients With Non-Small Cell Lung Cancer

**DOI:** 10.3389/fimmu.2021.670040

**Published:** 2021-08-26

**Authors:** Guangsheng Zhu, Ruifeng Shi, Yongwen Li, Zihe Zhang, Songlin Xu, Chen Chen, Peijun Cao, Hongbing Zhang, Minghui Liu, Zhenhua Pan, Hongyu Liu, Jun Chen

**Affiliations:** ^1^Department of Lung Cancer Surgery, Tianjin Medical University General Hospital, Tianjin, China; ^2^Tianjin Lung Cancer Institute, Tianjin Key Laboratory of Lung Cancer Metastasis and Tumor Microenvironment, Tianjin Medical University General Hospital, Tianjin, China; ^3^Quantitative Biomedical Research Center, Department of Population and Data Sciences, University of Texas Southwestern Medical Center, Dallas, TX, United States

**Keywords:** non-small cell lung cancer, immunotherapy, SWI/SNF complex, PD-1/PD-L1 inhibitors, anti-PD1/PD-L1

## Abstract

Worldwide, non-small cell lung cancer (NSCLC) has the highest morbidity and mortality of all malignancies. The lack of responsiveness to checkpoint inhibitors is a central problem in the modern era of cancer immunotherapy, with the rapid development of immune checkpoint inhibitors (ICIs) in recent years. The human switch/sucrose nonfermentable (SWI/SNF) chromatin-remodeling complex has been reported to be recurrently mutated in patients with cancer, and those with SWI/SNF mutations have been reported to be sensitive to ICIs. Six reported cohorts, a total of 3416 patients, were used to analyze the mutation status of ARID1A, ARID1B, ARID2 and SMARCA4 in patients with NSCLC and the effect of mutations on prognosis after ICIs. Finally, a nomogram was established to guide the clinical use of ICIs. The results show that patients with NSCLC who have ARID1A, ARID1B, and ARID2 mutations of the SWI/SNF complex were more likely to benefit from ICI therapy.

## Introduction

Lung cancer has the highest morbidity and mortality of all malignancies worldwide, with 80% - 85% of histological types diagnosed as non-small cell lung cancer (NSCLC). According to cancer statistics, worldwide, 9.6 million cancer deaths occurred in 2018, of which lung cancer showed the highest incidence and mortality (1). Recently, advances in understanding the complex relationship between tumor cells and the immune response have resulted in a paradigm shift in cancer immunology, and new and more effective approaches to cancer immunotherapy. Immune checkpoint inhibitors (ICIs), such as programmed cell death 1/programmed death ligand 1 (PD-1/PD-L1) and cytotoxic T-lymphocyte-associated protein 4 (CTLA-4) blockade, enable the adaptive immune response to recognize and kill tumor cells, revolutionizing the standard of care for several cancers, including NSCLC. Several clinical trials have shown that ICI therapy is effective for first- and second-line treatments of advanced NSCLC, consolidated treatment of locally advanced NSCLC, and neoadjuvant treatment of early NSCLC. However, despite the promising efficacy of immunotherapy in NSCLC, the success of ICIs is currently limited to a small subset of patients, with the overall response rate to anti–PD-1 or PD-L1 therapy only 20%- 30% (2, 3). Thus, strategies are needed to identify the most suitable candidates for ICIs. To date, several clinical predictors of the ICI response in NSCLC have been identified (e.g., mutational and neoantigen loads, and PDL-1 expression), with PD-L1 expression being used in clinical practice to select patients for therapy. However, the quantitative detection of PD-L1 as a prediction index requires antibodies and staining platforms, which contribute to differences in the accuracy of PD-L1 levels, which may affect the predictive value. Moreover, clinical trials have shown that second-line treatment with anti–PD-1 or anti-PD-L1 antibodies may even be effective in patients with no PD-L1 expression on their tumor or immune cells (4), whereas patients with high PD-L1 expression sometimes fail to respond to anti–PD-1/PD-L1 therapy (5). The tumor mutation burden (TMB) (6), the total number of mutations per megabase in the coding regions of tumor cells, and neoantigen load, which indicate the neoantigens produced by tumor cells to active T cells, are other predictors of therapeutic efficacy. Some researchers have found that a high TMB and neoantigen load are associated with an improved response to ICI treatment (6–8), whereas others found no significant difference (9–11). Therefore, the establishment of new predictors to identify suitable candidates for immunotherapy is a central challenge in the modern era of cancer immunotherapy.

The human switch/sucrose nonfermentable (SWI/SNF) chromatin-remodeling complex is encoded by multi-gene families recurrently mutated in cancer. Previous studies have shown that tumors, such as renal clear cell carcinoma, harboring SWI/SNF mutations are sensitive to ICIs. Meanwhile, mutations in SWI/SNF complex genes, such as *SMARCA4*, *ARID1A*, *ARID1B*, and *ARID2*, affect the clinical outcomes of ICI treatments in patients with NSCLC. However, studies on the role of mutations of the SWI/SNF complex in ICI therapy for patients with NSCLC are lacking

In this study, publicly available profiles were collected and integrated, and a comprehensive analysis was performed to investigate the role of SWI/SNF complex gene mutations in the prognosis of patients with NSCLC treated with anti-PD-1/PD-L1 ICIs.

## Methods

### Data Sources

Whole-exome sequencing (WES) data of 1144 NSCLC cases from The Cancer Genome Atlas (TCGA) cohort (12) was obtained through cBioPortal (http://www.cbioportal.org/). The RNA-seq data of 515 LUAD and 501 LUSC were downloaded from the TCGA (https://portal.gdc.cancer.gov/). Five available clinical cohorts with 2272 patients who underwent ICI therapy at the Memorial Sloan Kettering Cancer Center (MSKCC) (9, 13–16) were included in this study. Detailed information for each cohort is shown in **Table 1**. Neoantigen data were obtained using a tumor immunograph network (https://tcia.at/home) (17). Tumor-infiltrating lymphocytes based on RNA-sequencing (seq) data were obtained from TIMER (http://timer.comp-genomics.org/) (18).

**Table 1:** Baseline data of 3416 patients with non-small cell lung cancer.

Characteristic		TCGA Cohort n=1144	Zehir Cohort n=1567	Samstein Cohort n=355	Hellmann Cohort n=75	Rizvi Cohort n=240	Naiyer Cohort n=35
Gender	Male	673 (59%)	681 (43%)	166 (48%)	37 (49%)	118 (49%)	16 (46%)
	Female	468 (41%)	886 (57%)	178 (52%)	38 (51%)	122 (51%)	19 (54%)
Age	>60	695 (71%)	NA	246 (72%)	47 (63%)	156 (65%)	19 (54%)
	<=60	253 (26%)	NA	98 (28%)	28 (37%)	86 (35%)	16 (46%)
Smoking status	Ever	976 (85%)	972 (62%)	NA	60 (80%)	197 (80%)	30 (86%)
	Never	111 (10%)	334 (21%)	NA	15 (20%)	47 (20%)	5 (14%)
	Unknown	57 (5%)	261 (17%)	NA	0	0	0
Histology	AD	660 (58%)	1268 (81%)	268 (78%)	Non-SCC:59 (79%)	186 (78%)	30 (86%)
	SCC	484 (42%)	163 (10%)	44 (13%)	16 (21%)	34 (14%)	4 (11%)
	Others	0	136 (9%)	30 (9%)	0	20 (8%)	1 (3%)
Treatment type	PD1/PDL1	NA	NA	324 (91%)	0	206 (86%)	35 (100%)
	CTLA4	NA	NA	0	0	0	0
	Combo	NA	NA	20 (6%)	75 (100%)	34 (14%)	0
PDL1	PDL1	0	NA	NA	70 (93%)	86 (36%)	30 (86%)
	CD274	1015 (89%)	NA	NA	0	0	0
Neoantigen		1053 (92%)	NA	NA	75 (100%)	NA	NA

NA, Not Available.

### Assessment of the TMB

Mutation profiles were assessed by WES in Hellmann (14), Naiyer (16), and TCGA cohorts and by next-generation sequencing in Zehir (13), Rizvi (15), and Samstein (9) cohorts. The TMB is the number of gene synonymous variants per million base-pairs detected in tumor tissue. The TMB was defined as the number of non-silent somatic mutation counts in coding regions. A TMB-low population was defined as patients with <10 mut/MB (9).

### Messenger RNA Expression Profiling Analysis of Immune-Related Signatures

Tumor immune microenvironment-related signatures, including chemokines, chemokine receptors, immunostimulators, and immunoinhibitors, were compared. Associations between SWI/SNF complex gene mutations and relevant immune-related genes were analyzed in 1016 patients from the TCGA cohort, for whom both RNA-seq and DNA-seq data were available. The list of immune genes was mainly based on published articles that summarized genes related to immunotherapy. The list of 63 immune genes is provided in **Supplementary Table 1**.

### Construction of an Integrated Prognostic Classifier Model

As shown in **Supplementary Table 2**, univariate Cox regression analysis was used to screen for factors significantly associated with progression-free survival (PFS). Smoking history, treatment type (anti–PD-1/PD-L1 or anti-CTLA4), PD-L1 immunohistochemistry (IHC) score, TMB, SWI/SNF mutation status, and epidermal growth factor receptor (EGFR) mutation status were included for further analysis of the Rizvi cohort. A multivariate Cox regression analysis model was constructed using elected factors and “rms”, “foreign”, “survival”, “tidyverse”, and “survivalROC” packages of R. A calibration curve of the nomogram was made for internal verification. The risk score was calculated according to its regression coefficient, and patients were divided into low- and high-risk score groups according to the cutoff value. The Naiyer cohort was used as an external validation cohort to validate the model.

### Statistical Analyses

Statistical analyses were performed using R version 3.6.2. The packages: “ggplot2”, “rms”, “foreign”, “survivalROC”, and “survival” were used for statistical and graphics analyses, and the packages “survival” and “survminer” were used for survival analysis. Pearson’s correlation coefficient was used to analyze the correlation between two continuous variables. An independent sample *t*-test was used to compare two groups of samples. The Wilcoxon test was used to compare multiple groups of samples, and the log-rank test was used to compare two or more survival curves. *P* < 0.05 was considered statistically significant. The Benjamin & Hochberg method was used to adjust the *P* value.

## Results

### Demographic and Clinical Characteristics of the Study Cohorts

Basic information of the six cohorts is shown in **Table 1**. A total of 3416 patients, 1711 females and 1691 males, were included in this study. The study participants comprised 2412 patients with adenocarcinoma and 745 patients with squamous carcinoma; 2235 patients were smokers and 512 were non-smokers, and the median age was 61 years. **Table 1** shows the demographic and clinical characteristics of the study cohorts.

### SWI/SNF Complex Genes Were Frequently Mutated in Patients With NSCLC

**Figure 1:**
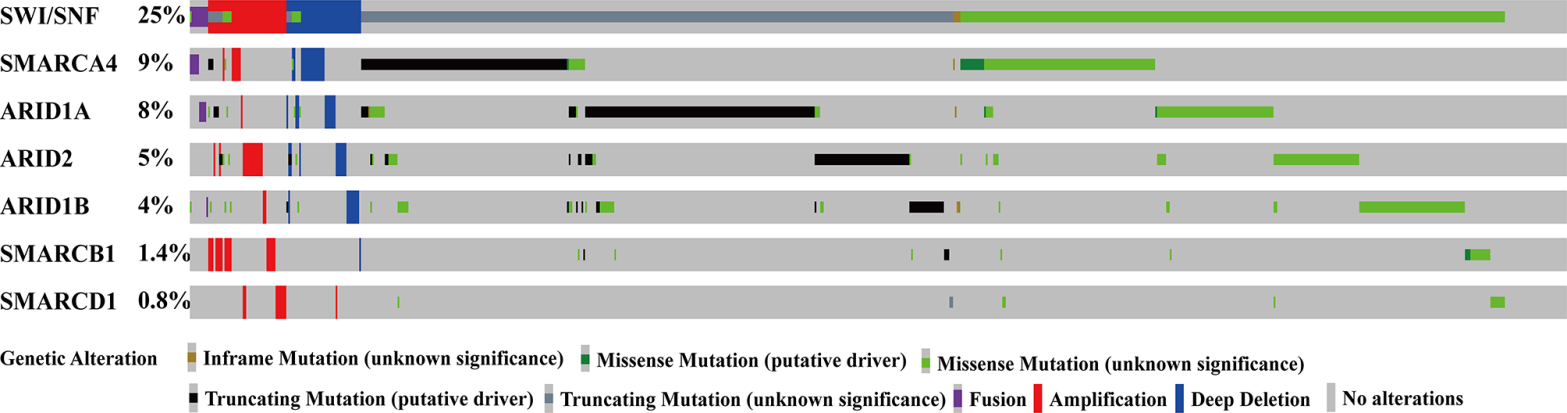
SWI/SNF complex genes were frequently mutated in patients with NSCLC. SWI/SNF, human switch/sucrose nonfermentable; NSCLC, non-small cell lung cancer.

Of the 3416 NSCLC patients, approximately 25% had at least one SWI/SNF complex gene mutation; of these, 9% harbored *SMARCA4* mutations, 8% harbored *ARID1A* mutations, 5% harbored *ARID2* mutations, and 4% harbored *ARID1B* mutations. **Figure 1** shows detailed mutations for each gene.

Additionally, SWI/SNF complex gene mutations rarely occurred simultaneously with V-Ki-ras2 Kirsten ratsarcoma viral oncogene homolog (KRAS) and EGFR mutations (**Supplementary Figure 1**).

The association between mutations of SWI/SNF complex genes with demographic and clinical factors, such as sex, age, smoking status, histology, and distant metastasis, was analyzed. Mutations in SWI/SNF complex genes were found to be significantly frequent in smokers, indicating that tobacco exposure may significantly impact mutations in the SWI/SNF complex. Additionally, *ARID1A* and *ARID2* mutations were more frequently found in males, *SMARCA4* was more frequently mutated in patients with adenocarcinoma, and the *ARID1B* mutation was more frequently found in patients with squamous carcinoma, which were all statistically significant (**Table 2**).

**Table 2:** Correlation analysis of SMARCA4,ARID1A,ARID1B,ARID2,SMARCB1,SMARCD1 gene mutations with gender, pathological status, age, smoking status, whether distal metastasis.

Characteristic		Gender		Histology			Age		Smoking Status		Metastasis	
		Man	Woman	AD	SCC	Other	>60	<=60	Yes	No	Yes	No
SMARCA4	Wild	1386	1439	1944	683	157	714	267	2066	483	224	683
	Mutation	140	120	202	24	20	49	27	212	21	13	44
	P	0.140		<0.001			0.119		<0.001		0.871	
ARID1A	Wild	1399	1454	1994	651	150	723	273	2085	482	223	685
	Mutation	133	105	152	56	27	40	21	193	22	14	42
	P	0.039		0.457			0.235		0.002		1	
ARID2	Wild	1430	1495	2024	677	171	724	277	2149	483	221	691
	Mutation	96	64	122	30	6	39	17	129	21	16	36
	P	0.006		0.139			0.663		0.178		0.369	
ARID1B	Wild	1470	1488	2076	664	170	721	272	2172	495	225	687
	Mutation	56	71	70	43	7	42	22	106	9	12	40
	P	0.216		0.001			0.227		0.003		0.925	
SMARCB1	Wild	1506	1549	2130	703	173	754	290	2254	500	233	724
	Mutation	20	10	16	4	4	9	4	24	4	4	3
	p	0.058		0.619			0.811		0.597		0.117	
SMARCD1	Wild	1516	1550	2133	703	175	737	278	2261	503	236	722
	Mutation	10	9	13	4	2	5	3	17	1	1	5
	P	0.782		0.905			0.539		0.165		1	

### *ARID1A*, *ARID1B*, and *ARID2* Mutations Are Associated With Better Outcomes for Patients With NSCLC Treated With ICIs

No significant difference in PFS and overall survival (OS) was observed between wild-type (WT) and *SMARCA4* mutation groups in either Samstein or Hellmann, Rizvi, and Naiyer (HRN) cohorts. However, patients with *ARID1B* mutations had a better median PFS [mPFS; 22.4 *vs.* 4; hazard ratio (HR) = 0.442; 95% confidence interval (95% CI) = 0.235–0.833; *P* = 0.0092; **Supplementary Figure 2A**]. Patients with *ARID1A*, *ARID1B*, and *ARID2* mutations had better OS, although the difference was not significant; this may be due to the limited number of cases with mutations (**Supplementary Figure 1B**). Patients with an *ARID1A* or *ARID1B* mutation treated with ICIs had a median OS (mOS) of 21 months compared to 11 months for the WT group. Patients with an *ARID2* mutation treated with ICIs had an mOS of 36 months compared to 11 months for the WT group (**Supplementary Figure 2B**).

**Figure 2:**
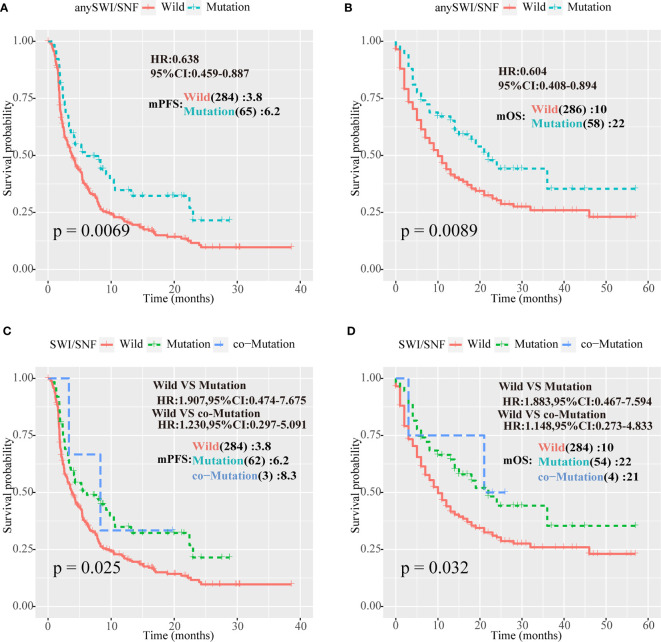
Human switch/sucrose nonfermentable (SWI/SNF) complex mutations were associated with better outcomes for patients with NSCLC treated with PD-1/PD-L1 inhibitors. **(A, B)** Survival curves of progression-free survival (PFS) for the Hellmann, Rizvi, and Naiyer (HRN) cohort, and of overall survival (OS) for the Samstein cohort according to *ARID1A*, *ARID1B*, and *ARID2* mutations in patients with non-small cell lung cancer (NSCLC). Patients with at least one mutation in one of the three genes were part of the human switch/sucrose nonfermentable (SWI/SNF) complex mutation group, and the remaining patients were part of the wild-type (WT) group. **(C, D)** PFS curve for the Rizvi cohort and OS for the Samstein cohort according to *ARID1A*, *ARID1B*, and *ARID2* mutations in NSCLC patients. Patients with no mutations in any of the three genes formed the WT group, patients with one mutation were part of the one-mutation group, and patients with two or more mutations formed the co-mutation group.

*ARID1A*, *ARID1B*, and *ARID2* analyses were combined. Patients with at least one mutation in one of the three genes were defined as the SWI/SNF complex mutation group, and the remaining patients were defined as the WT group. In a survival analysis of the two cohorts, for the HRN cohort, the mPFS of the mutant and WT groups was 6.2 *vs.* 3.8 months, respectively (*P* = 0.0069; HR = 0.638; 95% CI = 0.459–0.887; **Figure 2A**), whereas the mOS of the mutant and WT groups was 22 *vs.* 10 months, respectively (*P* = 0.0089; HR = 0.604; 95% CI = 0.408–0.894) in the Samstein cohort (**Figure 2B**).

We suspected that the increase in the number of cumulative mutations in SWI/SNF complex genes would improve immunotherapy efficacy. Therefore, patients without SWI/SNF complex gene mutations were defined as the WT group, patients with one mutation were defined as the one-mutation group, and patients with two or more mutations were defined as the co-mutation group. Survival analysis of the HRN and Samstein cohorts demonstrated that the mPFS values in the HRN cohort of the one-mutation and WT groups were 6.2 and 3.8 months, respectively (*P* = 0.025; HR = 1.907; 95% CI = 0.474–7.675; **Figure 2C**), whereas mOS in the Samstein cohort of the one-mutation and WT groups was 22 vs. 10 months, respectively (*P* = 0.032; HR = 1.883; 95% CI = 0.467–7.594) in the Samstein cohort (**Figure 2D**). However, due to the small number of patients, the co-mutation group only showed a better mPFS or mOS than the mutation group in the HRN cohort (**Figures 2C, D**).

### Tendency of Patients With SWI/SNF Mutations to Have High TMB and Neoantigen Loads

In the Rizvi and Hellmann groups, higher PD-L1 IHC scores were observed in the any *SWI/SNF* and *ARID1B* mutation groups, and lower PD-L1 IHC scores were observed in the *SMARCA4* mutation group (**Supplementary Figure 3A**).

In patients with NSCLC and low PD-L1 scores (<50), the mPFS of patients with any SWI/SNF complex mutation was superior to that of WT patients treated with ICIs (8.3 *vs.* 3.7 months; *P* = 0.001; HR = 0.420; 95% CI = 0.246–0.717; **Supplementary Figure 3B**).

**Figure 3:**
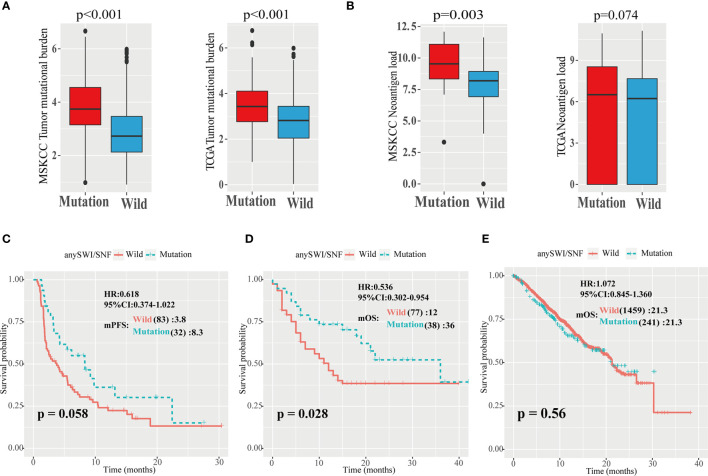
High TMB and neoantigen load of patients with SWI/SNF mutations. **(A)** Analysis of tumor mutation burden (TMB) values in five independent Memorial Sloan Kettering Cancer Center (MSKCC) cohorts, including the Zehir, Samstein, Rizvi, Naiyer, and Hellmann cohorts (left), and The Cancer Genome Atlas (TCGA) cohort (right). **(B)** Analysis of neoantigen load in Hellmann (left) and TCGA (right) cohorts. **(C, D) **Progression-free survival (PFS) curves of patients with non-small cell lung cancer (NSCLC) in the TMB-high group of the Hellmann, Rizvi, and Naiyer (HRN) **(C)** and Samstein **(D)** cohorts based on *ARID1A*, *ARID1B*, and *ARID2* mutations. SWI/SNF, human switch/sucrose nonfermentable. **(E)** Overall survival (OS) curves of patients with non-small cell lung cancer (NSCLC) in the Zehir cohort based on the human switch/sucrose nonfermentable (SWI/SNF) mutation status.

In the Zehir, Samstein, Rizvi, and Hellmann cohorts, the TMB of patients with *ARID1A*, *ARID1B*, and *ARID2* mutations of the SWI/SNF complex mutation group was significantly higher than that of the WT group (*P* < 0.001; **Figure 3A**, left). Similarly, in the TCGA cohort, the TMB of patients with *ARID1A*, *ARID1B*, and *ARID2* gene mutations was significantly higher than that of the WT group (*P* < 0.001; **Figure 3A**, right).

In TMB-high (>10) patients with NSCLC, the mPFS of patients with any SWI/SNF complex mutation was superior to that of WT patients (8.3 *vs.* 3.8 months; *P* = 0.058; HR = 0.618; 95% CI = 0.374–1.022; **Figure 3C**). In TMB-high patients with NSCLC in the Samstein cohort, the mOS of patients with any SWI/SNF complex mutation was significantly superior to that of WT patients (36 *vs.* 12 months; *P* = 0.028; HR = 0.536; 95% CI = 0.302–0.954; **Figure 3D**). There was no significant difference between any SWI/SNF mutation and WT subgroups in the mPFS or mOS of TMB-low patients with NSCLC in the two cohorts (**Supplementary Figures 3C, D**). Moreover, in the non-ICIs treated NSCLC population, the mutations of the SWI/SNF complex did not have a better survival benefit (**Figure 3E**). Therefore, SWI/SNF is a prognostic indicator and a true predictor independent on PD-L1 and TMB.

The relationship between neoantigen load and SWI/SNF complex mutations was also explored. It was found that patients with any SWI/SNF complex gene mutation had elevated neoantigen loads (*P* = 0.003; **Figure 3B**).

### Decreased Activated Dendritic Cells and Monocyte Infiltration, and Altered Immune Microenvironment, in NSCLC Patients With *ARID1A*, *ARID1B*, or *ARID2* Mutation

**Figure 4:**
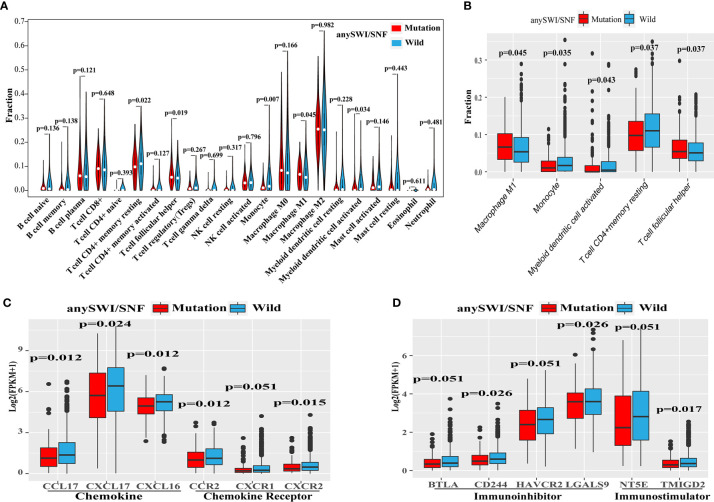
Altered immune microenvironment in SWI/SNF mutation patients. **(A)** Violin plot of the relative infiltration of 22 immune cell types in The Cancer Genome Atlas (TCGA) cohort. **(B)** Immune infiltration of monocytes or dendritic cells according to the human switch/sucrose nonfermentable (SWI/SNF) complex mutation status of the TCGA cohort. **(C, D)** Expression of chemokines or chemokine receptors **(C)** and immunoinhibitors or immunostimulators **(D)** according to the SWI/SNF complex mutation status of the TCGA cohort.

To investigate correlations between the infiltration of immune cells and SWI/SNF complex gene mutations, 22 immune cell types were analyzed using expression data from the TCGA dataset. The immune infiltration levels of monocytes, myeloid dendritic cell activated, and T-cell CD4^+^ memory resting cells were decreased in patients with an *ARID1A*, *ARID1B*, or *ARID2* mutation. However, macrophage M1 and T-cell follicular helper cell levels were increased in patients with an *ARID1A*, *ARID1B*, or *ARID2* mutation (**Figures 4A, B**).

The expression levels of chemokines, chemokine receptors, immunoinhibitors, and immunostimulators were also analyzed to further explore whether SWI/SNF complex mutations affect the expression of immune-related cytokines (**Supplementary Table 1**). Patients with SWI/SNF complex gene mutations were found to have lower expression levels of the following gene clusters: chemokines (CCL17, CXCL17, and CXCL16; **Figure 4C**), chemokine receptors (CXCR2, CXCR1, and CCR2; **Figure 4C**), immunoinhibitors (BTLA, CD244, HAVCR2, and LGALS9; **Figure 4D**), and immunostimulators (NT5E and TMIGD2; **Figure 4D**).

### Construction of an Integrated Prognostic Classifier Model for Predicting the Efficacy of ICI Therapy

**Figure 5:**
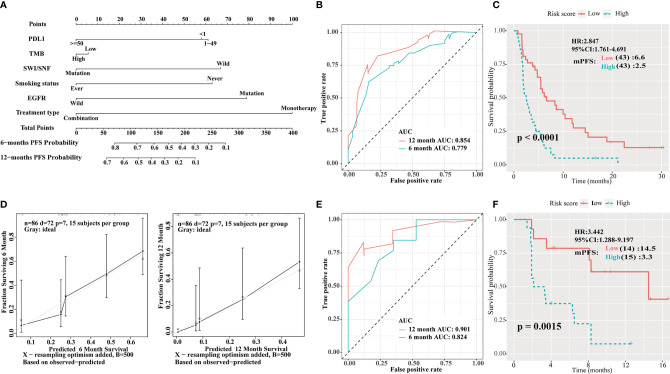
Construction of an integrated prognostic classifier model. **(A)** Nomogram based on programmed death ligand 1 (PD-L1) score, tumor mutation burden (TMB), human switch/sucrose nonfermentable (SWI/SNF) mutation status, smoking history, epidermal growth factor receptor (EGFR) mutation status, and treatment type of the Rizvi cohort. **(B)** Receiver operating characteristic (ROC) curves for predicting progression-free survival (PFS) of the nomogram in the Rizvi cohort. **(C)** Calibration plot of the nomogram for the probability of PFS at 6 (left) and 12 (right) months in the Rizvi cohort. **(D)** Survival curve of PFS with the nomogram in the Rizvi cohort. The risk score was calculated according to the regression coefficient. The cohort was divided into low- and high-risk score groups for Kaplan-Meier curve analysis. **(E)** ROC curves for predicting PFS of the nomogram in the Naiyer cohort. **(F)** Survival curve of PFS with the nomogram according to the risk score in the Naiyer cohort.

Univariate analysis showed that PD-L1 score, TMB, SWI/SNF mutation status, smoking history, EGFR mutation status and treatment type, were statistically significant in predicting PFS in the Rizvi cohort. A nomogram was then developed to predict 6- and 12-month PFS using the above six factors in the Rizvi cohort (**Figure 5A**). Receiver operating characteristic (ROC) analysis indicated good accuracy of this model (area under the curve [AUC] of 6-month survival, 0.779; AUC of 12-month survival, 0.854; **Figure 5B**); the calibration curve also suggested an acceptable accuracy (**Figure 5D**). The PFS survival curve showed that the low-risk group had a better mPFS than the high-risk group (6.6 *vs.* 2.5; *P* < 0.001; HR = 2.847; 95% CI = 1.761–4.691; **Figure 5C**). Furthermore, the Naiyer cohort was used as an external validation cohort to verify the prognostic value of this immune signature. The ROC curve suggested that this immune signature was highly consistent with the ideal model (AUC of 6-month survival, 0.824; AUC of 12-month survival, 0.901; **Figure 5E**). The PFS survival curve showed that the low-risk group also had a better mPFS than the high-risk group (14.5 *vs*. 3.3; *P* = 0.0015; HR = 3.442; 95% CI = 1.288–9.197; **Figure 5F**).

## Discussion

The lack of responsiveness to checkpoint inhibitors is a central problem in the modern era of cancer immunotherapy. At present, a PD-L1 score measured by IHC is the standard predictive biomarker for anti-PD-1/PD-L1 ICI therapy. However, clinical trials have shown the deficiency of this biomarker as a predictor of such therapy (2, 3).

In this study, SWI/SNF complex genes were frequently mutated in patients with NSCLC. Furthermore, patients with NSCLC treated with PD-1/PD-L1/CTLA-4 inhibitors and having *ARID1A*, *ARID1B*, or *ARID2* mutations of the SWI/SNF complex showed better outcomes in comparison to those without such mutations. The mOS of patients with at least one of these mutations was 22 months compared to 10 months for the WT group (*P* = 0.0089; HR = 0.604; 95% CI = 0.408–0.894; **Figure 2B**), whereas the mPFS of patients with at least one of these mutations was 6.2 *vs* 3.8 months for the WT group (*P* = 0.0069; HR = 0.638; 95% CI = 0.459–0.887; **Figure 2A**). Additionally, cumulative mutations of the SWI/SNF complex were beneficial to the efficacy of ICI therapy. The mPFS for the co-mutation group was 8.3 months compared to 3.8 months for the WT group (**Figure 2C**). Moreover, in the non-ICIs-treated NSCLC population, the mutations of the SWI/SNF complex did not have a better survival benefit (**Figure 3E**). This indicates that the SWI/SNF complex mutation has a survival benefit for NSCLC patients treated with ICIs. Furthermore, a comprehensive predictive classifier model was built to evaluate the efficacy of ICI therapy according to SWI/SNF mutation status and clinical factors, such as smoking history, treatment type, PD-L1 score, and TMB. ROC curves for 6 and 12 months were drawn. AUCs were calculated as 0.779 and 0.854 for the test cohort, and 0.824 and 0.901 for the validation cohort, respectively. The risk score was calculated according to the regression coefficient. The low-risk group showed better mPFS (2.5 *vs.* 6.6; *P* < 0.001; HR = 2.847; 95% CI = 1.761–4.691 for the Rizvi cohort; **Figure 5D** and 3.3 *vs*. 14.5; *P* = 0.0015; HR = 3.442; 95% CI = 1.288–9.197 for the Naiyer cohort; **Figure 5F**). These results revealed the roles of *ARID1A*, *ARID1B*, and *ARID2* mutations in predicting the outcome for patients with NSCLC treated with ICIs. These findings indicated that a comprehensive model, including SWI/SNF complex mutation status and other clinical factors, will guide the use of immunotherapy and provide a reference for individualized immunotherapy against NSCLC.

The central function of the SWI/SNF complex is the coordinated regulation of gene expression programs by remodeling chromatin structure and regulating transcription by remodeling nucleosome occupancy at critical DNA elements. To investigate whether mutations of the SWI/SNF complex can influence the expression of PD-L1, scores for PD-L1 were compared between datasets from Rizvi and Hellmann cohorts, in which PD-L1 scores were available from IHC assays. PD-L1 mRNA expression levels were also compared to the TCGA dataset, in which PD-L1 RNA-sequencing data were available. Higher PD-L1 scores were observed in the *ARIDA1B* mutation group and lower PD-L1 scores were observed in the *SMARCA4* mutation group, with no significant difference in mRNA expression in the TCGA cohort. Further investigation will help reveal whether the SWI/SNF complex is involved in the regulation of PD-L1 and thus whether it plays a role in mediating immune escape in the context of lung cancer.

In this study, *ARID1A*, *ARID1B*, and *ARID2* gene mutations of the SWI/SNF complex were associated with increased TMB and neoantigen load. TMB, the total number of mutations per megabase in the coding regions of tumor cells, reflects the instability of tumor cells (8, 19). Because the activation of adaptive immunity requires antigen recognition, increased antigen recognition indicates a greater immune response. A high TMB may indicate that more neoantigens can be produced by tumor cells to activate T cells suppressed by immune checkpoint molecules. As increased TMB is associated with increased neoantigen load, this is usually associated with greater immunogenicity and a stronger immune response (19). Furthermore, our study also revealed that although a difference between any SWI/SNF mutation and WT subgroups was not apparent in terms of mPFS or mOS in TMB-low patients with NSCLC, in TMB-high patients, the mPFS or mOS of patients with *ARID1A*, *ARID1B*, or *ARID2* mutations was superior to those of WT patients (8.3 *vs*. 3.8 months; *P* = 0.058; HR = 0.618; 95% CI = 0.374–1.022 for PFS; and 36 *vs.* 12 months; *P* = 0.028; HR = 0.536; 95% CI = 0.302–0.954 for OS). These results indicated that, in TMB-high patients, *ARID1A*, *ARID1B*, and *ARID2* mutations indeed enhanced the immune response to PD-1/PD-L1 blockade. Additionally, the mPFS of PD-L1-low patients with at least one of these mutations was 8.3 months compared to 3.7 months for the WT group (*P* = 0.001; HR = 0.420; 95% CI = 0.246–0.7170; **Supplementary Figure 3C**). The mOS of TMB-high patients with at least one of these mutations was 36 months compared to 12 months for the WT group (*P* = 0.028; HR = 0.536; 95% CI = 0.302–0.954; **Figure 3D**).

These results also indicated that the immune microenvironment was altered in NSCLC patients who had *ARID1A*, *ARID1B*, or *ARID2* mutations. Compared with patients of the WT group, patients with mutations showed decreased the percentage of M1 macrophages, T helper cells, resting memory CD4+ T cells, monocytes and activated dendritic cells. In previous reports, the increased infiltration of M1 macrophages and follicular T helper cells is related to the better prognosis of lung cancer (20, 21). Meanwhile, the activation of resting memory CD4+ T cells has been reported to contribute to the progression and development of lung adenocarcinoma (22). Monocytes have also been reported as immunosuppressive cells in small cell lung cancer (23). Presently, there is still a lack of research on the relationship between the above cell infiltration and SWI/SNF complex. Moreover, the expression of chemokines (CCL17, CXCL17, and CXCL16), chemokine receptors (CXCR2, CXCR1, and CCR2), immunoinhibitors (BTLA, CD244, HAVCR2, and LGALS9), and immunostimulators (NT5E and TMIGD2) was reduced (**Figure 4C, D**). Cytokines play an important role in the differentiation, maturation, and migration of various immune cells (24, 25). CCL17, CXCR2, LGALS9 and NT5E recruits regulatory T cells into tumors as a mechanism of anti-tumor immune impairment (26–29). CXCL17 induces immature myeloid dendritic cells to infiltrate human pancreatic cancer, thereby promoting the immune response (30, 31). CXCL16 also plays an important role in enhancing the immune function of breast cancer by attracting T cell infiltration (32). Meanwhile, monocytes recruited by CCR2 will increase the number of lung metastases in breast cancer (33). BTLA and HAVCR2 mediate the inhibition of human tumor specific CD8 + T cells (28, 34), and CD244 mediates the dysfunction of natural killer cells (35). The relationship between these genes and the SWI/SNF complex is still unknown. Activated T-cell recruitment to tumor sites is necessary to mediate tumor cell killing (33). The efficacy of anti-PD-1 immunotherapy can be predicted according to the degree of immune cell infiltration, as determined by chemokines and entry through tumor blood vessels (33, 36). Therefore, further investigation of the roles of the SWI/SNF complex and the immune microenvironment will help us understand the mechanism of PD-1/PD-L1 blockade.

## Conclusion

The results of this study demonstrated that patients with *ARID1A*, *ARID1B*, or *ARID2* mutations were more likely to benefit from ICIs. A clinical prognosis prediction model will help guide the use of immunotherapy in patients with NSCLC and provide a reference for individualized immunotherapy of NSCLC in the future.

## Data Availability Statement

The original contributions presented in the study are included in the article/**Supplementary Material**. Further inquiries can be directed to the corresponding author/s.

## Author Contributions

GZ, YL and RS wrote the main manuscript text. ZZ, SX, and CC prepared Figures. PC, HZ, ML, and ZP contributed to data analysis. HL and JC contributed to data acquisition. All authors contributed to the article and approved the submitted version.

## Funding

This study was supported by grants from the National Natural Science Foundation of China (82072595, 81773207 and 61973232), Natural Science Foundation of Tianjin (17YFZCSY00840, 18PTZWHZ00240, 19YFZCSY00040, and 19JCYBJC27000), Shihezi University Oasis Scholars Research Startup Project(LX202002), and the Special Support Program for the High-Tech Leader and Team of Tianjin (TJTZJH-GCCCXCYTD-2-6). Funding sources had no role in study design, data collection and analysis, in the decision to publish, or in the preparation of the manuscript.

## Conflict of Interest

The authors declare that the research was conducted in the absence of any commercial or financial relationships that could be construed as a potential conflict of interest.

## Publisher’s Note

All claims expressed in this article are solely those of the authors and do not necessarily represent those of their affiliated organizations, or those of the publisher, the editors and the reviewers. Any product that may be evaluated in this article, or claim that may be made by its manufacturer, is not guaranteed or endorsed by the publisher.

## Supplementary Material

Supplementary Figure 1 Human switch/sucrose nonfermentable (SWI/SNF) complex gene mutations rarely occurred simultaneously with KRAS and EGFR mutations.
