# Supplementary material for: ARID1A, ARID1B, and ARID2 Mutations Serve as Potential Biomarkers for Immune Checkpoint Blockade in Patients With Non-Small Cell Lung Cancer
Source: Front Immunol. 2021 Aug 26;12:670040. doi: 10.3389/fimmu.2021.670040 (PMC8426508; doi:10.3389/fimmu.2021.670040)
Supplement: Supplementary Figure 1 — Human switch/sucrose nonfermentable (SWI/SNF) complex gene mutations rarely occurred simultaneously with KRAS and EGFR mutations. [file DataSheet_1.docx]

Supplementary Material

# Supplementary Figures and Tables

## Supplementary Figures


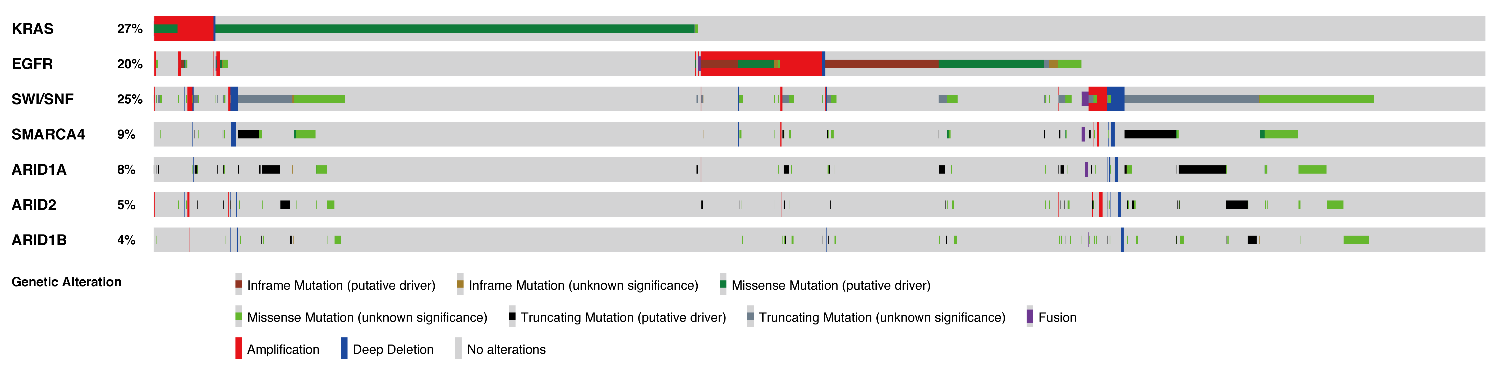


**Supplementary Figur**e 1. Human switch/sucrose nonfermentable (SWI/SNF) complex gene mutations rarely occurred simultaneously with KRAS and EGFR mutations.


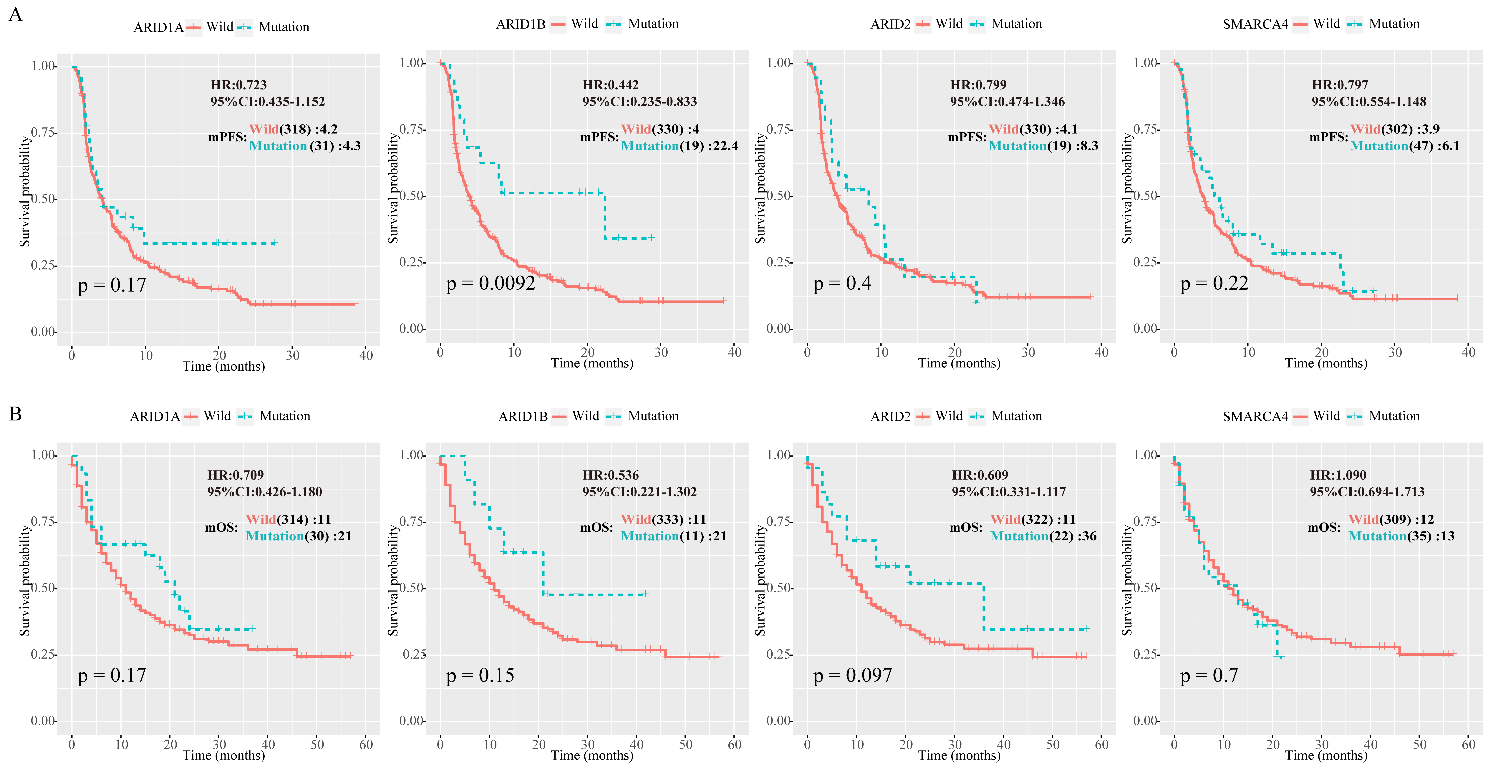


**Supplementary Figure 2.** *ARID1A*, *ARID1B*, and *ARID2* mutations were associated with better outcomes for patients with non-small cell lung cancer (NSCLC) treated with immune checkpoint inhibitors (ICIs). A: Progression-free survival (PFS) curve according to *SMARCA4*, *ARID1A*, *ARID1B*, and *ARID2* mutations in the cohort. B: Overall survival (OS) curve according to *SMARCA4*, *ARID1A*, *ARID1B*, and *ARID2* mutations.

**
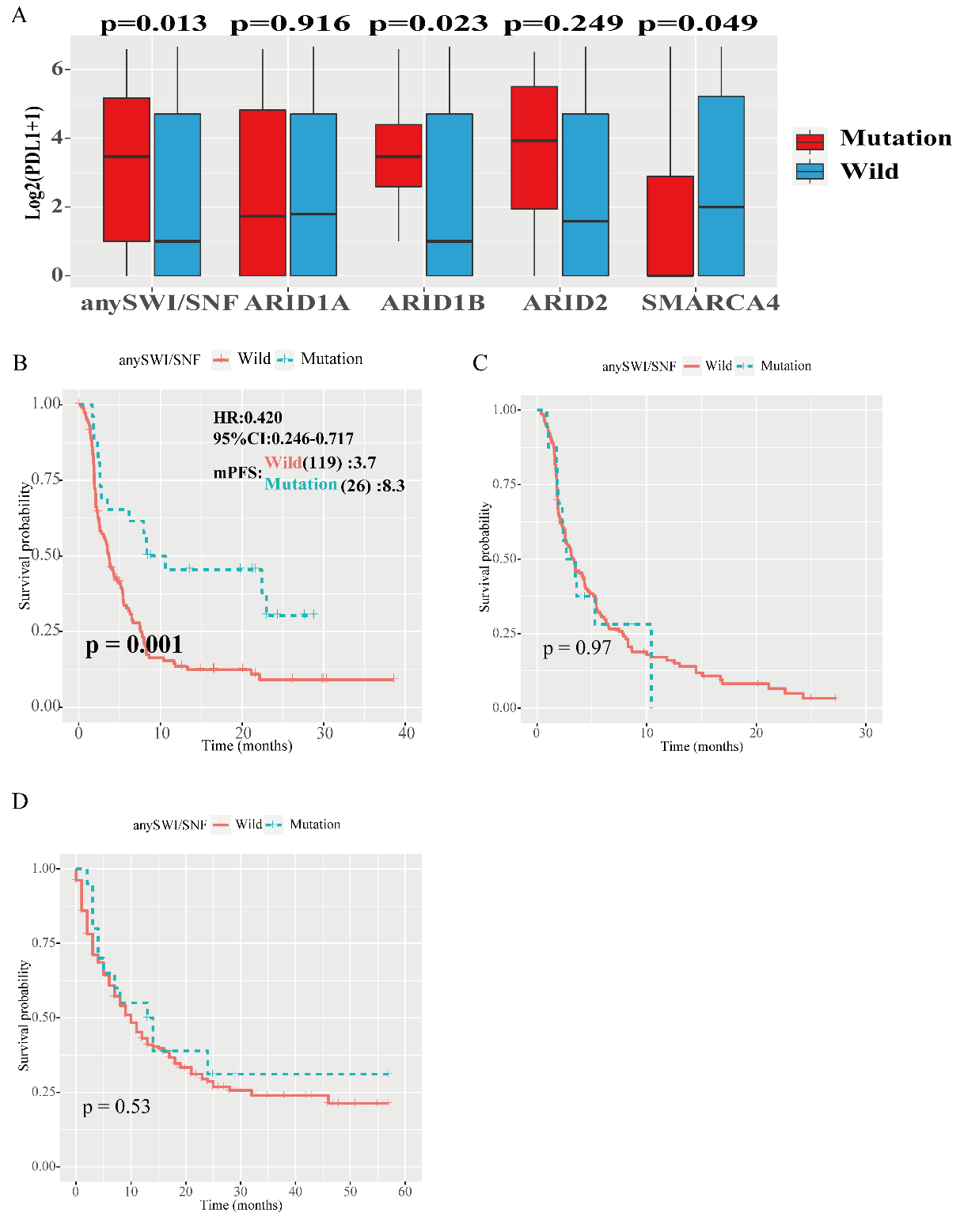
**

**Supplementary Figure 3.** (A) Analysis of programmed death ligand 1 (PD-L1) immunohistochemical (IHC) scores according to *SMARCA4*, *ARID1A*, *ARID1B*, and *ARID2* mutations in the Rizvi and Hellmann cohorts. (B) Progression-free survival (PFS) curves of patients with NSCLC in the programmed death ligand 1 (PD-L1)-low group of the HRN cohort. (C) PFS curves of patients with NSCLC in the Rizvi cohort based on SWI/SNF mutation status of TMB-low patients. (D) PFS curves of patients with NSCLC in the Samstein cohort based on the SWI/SNF mutation status of TMB-low patients.

## Supplementary Table

**Supplementary Table 1**: Immune related cytokines, cytokine receptors, immune stimulating genes, immunosuppressive genes.

| Chemokine | CCL5 |
| --- | --- |
| Chemokine | CCL7 |
| Chemokine | CCL14 |
| Chemokine | CCL17 |
| Chemokine | CCL19 |
| Chemokine | CCL23 |
| Chemokine | CCL24 |
| Chemokine | CCL26 |
| Chemokine | CXCL3 |
| Chemokine | CXCL8 |
| Chemokine | CXCL10 |
| Chemokine | CXCL13 |
| Chemokine | CXCL16 |
| Chemokine | CXCL17 |
| Chemokine receptor | CCR1 |
| Chemokine receptor | CCR2 |
| Chemokine receptor | CXCR1 |
| Chemokine receptor | CXCR2 |
| Chemokine receptor | CXCR3 |
| Chemokine receptor | CXCR6 |
| Chemokine receptor | XCR1 |
| Immunoinhibitor | ADORA2A |
| Immunoinhibitor | BTLA |
| Immunoinhibitor | CD244 |
| Immunoinhibitor | CD96 |
| Immunoinhibitor | HAVCR2 |
| Immunoinhibitor | IL10 |
| Immunoinhibitor | LAG3 |
| Immunoinhibitor | LGALS9 |
| Immunoinhibitor | VTCN1 |
| Immunostimulator | CD48 |
| Immunostimulator | CD80 |
| Immunostimulator | ENTPD1 |
| Immunostimulator | ICOSLG |
| Immunostimulator | KLRK1 |
| Immunostimulator | MICB |
| Immunostimulator | NT5E |
| Immunostimulator | PVR |
| Immunostimulator | TMIGD2 |
| Immunostimulator | TNFRSF14 |
| Immunostimulator | TNFRSF17 |
| Immunostimulator | TNFRSF9 |
| Immunostimulator | TNFSF14 |
| Immunostimulator | TNFSF15 |
| Immunostimulator | TNFSF18 |
| Immunostimulator | TNFSF4 |
| Immunostimulator | TNFSF9 |
| Immunostimulator | ULBP1 |

**Supplementary Table 2**: Univariate Cox regression analysis of clinical data in Rizvi cohort.

| ID | HR | HR.95L | HR.95H | *P* value |
| --- | --- | --- | --- | --- |
| Sex | 0.814514 | 0.51164 | 1.296677 | 0.387138 |
| Age | 0.954949 | 0.751363 | 1.213696 | 0.706306 |
| Smoke | 0.476618 | 0.273855 | 0.829508 | 0.008764 |
| SWI/SNF | 0.436316 | 0.2081 | 0.914808 | 0.028114 |
| PD-L1 | 0.646384 | 0.478493 | 0.873183 | 0.004459 |
| TMB | 0.587266 | 0.36309 | 0.94985 | 0.03003 |
| EGFR | 2.275344 | 1.119525 | 4.624451 | 0.023088 |
| KRAS | 0.932476 | 0.585668 | 1.484651 | 0.768286 |
| Treatment type | 0.374231 | 0.173856 | 0.805544 | 0.011978 |
